# What, If Anything, Is Rodent Prefrontal Cortex?

**DOI:** 10.1523/ENEURO.0315-18.2018

**Published:** 2018-10-25

**Authors:** Mark Laubach, Linda M. Amarante, Kyra Swanson, Samantha R. White

**Affiliations:** 1 Department of Biology and Center for Behavioral Neuroscience, American University, Washington, DC 20016

**Keywords:** atlas, callosum, cingulated, meta-analysis, prefrontal, survey

## Abstract

Prefrontal cortex (PFC) means different things to different people. In recent years, there has been a major increase in publications on the PFC, especially using mice. However, inconsistencies in the nomenclature and anatomical boundaries of PFC areas has made it difficult for researchers to compare data and interpret findings across species. We conducted a meta-analysis of publications on the PFC of humans and rodents and found dramatic differences in the focus of research on these species. In addition, we compared anatomical terms and criteria across several common rodent brain atlases and found inconsistencies among, and even within, leading atlases. To assess the impact of these issues on the research community, we conducted a survey of established PFC researchers on their use of anatomical terms and found little consensus. We report on the results of the survey and propose an alternative scheme for interpreting data from rodent studies, based on structural analysis of the corpus callosum and nomenclature used in research on the anterior cingulate cortex (ACC) of primates.

## Visual Abstract

**Figure:**
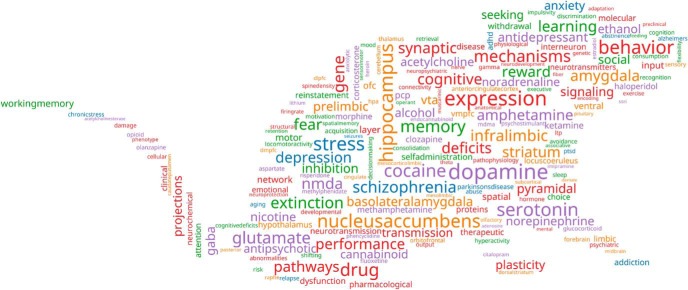

## Significance Statement

Studies on prefrontal parts of the rodent cerebral cortex have appeared at an increasing rate in recent years. However, there has been no consensus on the terms used to describe the rodent prefrontal cortex (PFC) or how it relates to the PFC of monkeys and humans. To address these issues, we conducted a meta-analysis of publications on the PFC across species, a review of rodent brain atlases, a survey of PFC researchers on anatomic terms, and an analysis of how species differences in the corpus callosum might help relate PFC areas across species. Addressing these issues may help improve the clarity, rigor, and reproducibility of research on the rodent PFC.

## 

The anatomic term “prefrontal” means different things to different people. For neuroscientists studying humans and nonhuman primates, prefrontal cortex (PFC) typically means the granular and orbital parts of the frontal cortex. These researchers often use variations of the term anterior cingulate cortex (ACC) to refer to agranular areas in the medial frontal cortex. By contrast, researchers studying rodents tend to use the term PFC to refer to the same medial frontal areas that are often called ACC in primates. Confusion can arise when the same anatomic term applies to different areas in different species. Anatomic terms should mean the same thing across species. The lack of consensus on terms and acronyms is apparent, for example, in the NeuroNames database, which seeks to account for anatomic terms across species that are commonly used in neuroscience research (Bowden et al., 2012). For the PFC, it has been difficult to incorporate findings made using the latest cutting-edge research tools, which typically depend on rodent research, into the larger PFC literature. It is easy to dismiss this issue as one of “mere” semantics or terminology, but it has significant, long-term consequences for our field. For instance, it is not uncommon for researchers to use information about one prefrontal area in a rodent species to understand a completely different prefrontal area in a primate species (Chen et al., 2013; Ferenczi and Deisseroth, 2016; Terraneo et al., 2016).

Here, we attempt to address this issue by asking a simple question: What, if anything, is rodent PFC? The phrase “if anything” emphasizes the idea that we are not sure if there is a clear answer to our question. In particular, we do not wish to dispute the idea that rodents have a PFC. However, it may be that a standard set of anatomic terms, based on established cross-species homologies in the frontal cortex (Vogt and Paxinos, 2014), should be used to describe the “rodent PFC.” These terms might or might not include the word “prefrontal” without implying that the area in question is not a prefrontal area. For example, researchers who study monkeys and humans know that the orbitofrontal cortex is part of the primate PFC without the need to include the word “prefrontal” in its name. That said, we are well aware of disagreements throughout the history of neuroscience about anatomic terms (e.g., V5 vs MT: Zeki, 2004) and the fact that there is no accepted “Code of Conduct” for reporting anatomic results. (Perhaps there should be.)

This article is in four parts. First, we review recent publication trends on PFC in humans, monkeys, rats, and mice. A meta-analysis reveals that human and rodent research has diverged to some extent, mostly due to a major focus of human studies on high-level cognitive processes that are associated with granular parts of the lateral PFC (Petrides, 2005). These areas do not exist in rodents (Preuss, 1995), and our meta-analysis reveals that rodent researchers have focused on cognitive functions associated with medial, but not lateral, PFC. Second, confusion and controversy about rodent PFC has persisted in part due to the use of multiple sets of anatomic terms and acronyms to describe areas on the medial wall of the frontal cortex and inconsistencies among, and even within, leading rodent brain atlases. We highlight some of these issues. Third, we report on a crowdsourcing effort that was conducted to assess usage of anatomic terms for the rodent PFC. The survey revealed that there is little consensus among researchers about what constitutes the rodent PFC and what terms should be used to describe this cortical region. Finally, we describe differences in the shape of the corpus callosum underlying the frontal cortex that might be helpful for comparing medial frontal areas across species. Our analysis suggests that rodent PFC areas may readily be understood within a framework that is commonly used to describe the primate ACC, and consists of dorsal, pregenual (rostral), and subgenual (ventral) regions with clearly dissociable connectivity and functions. To this end, we conclude with some recommendations for reporting anatomic data in rodent studies that should help resolve issues across labs and improve the rigor and reproducibility of PFC research.

## Publication Trends in Rodent PFC Research

**Figure 1.**
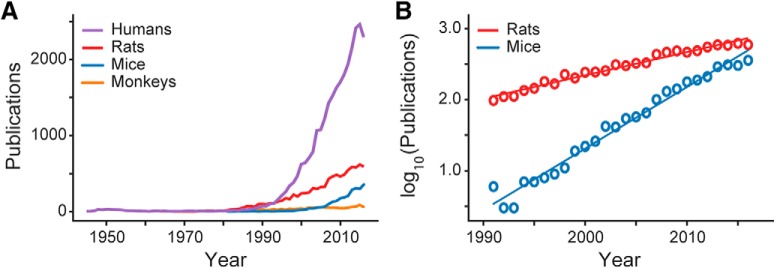
Publication trends in PFC research. ***A***, Publications per year on the PFC of humans, rats, mice, and monkeys from 1945 to 2016. Publication trends on monkey PFC (orange line) have remained unchanged and were not further analyzed. ***B***, Publications on the PFC of mice are appearing at a higher rate than those on rats in the period from 1990 to 2016.

Publications on the PFC have appeared at an increasing rate since 1990 (Fig. 1*A*). The sudden onset of interest in the PFC among neuroscientists was motivated by early studies on the PFC of macaque monkeys by research groups headed by Fuster (Fuster and Alexander, 1971) and Goldman-Rakic (for review, see Goldman-Rakic 1995). Key findings from these early studies were that neurons in the primate dorsolateral PFC (Brodmann’s areas 9 and 46) encode spatial “working memory” signals by firing persistently over delay periods (Funahashi et al., 1989) and that the signals were highly sensitive to perturbations of cortical dopamine receptors (Williams and Goldman-Rakic, 1995). In parallel with these neurophysiological studies, early neuroimaging studies (PET and fMRI) implicated the human PFC in higher-order cognitive processing such as attention (Pardo et al., 1991) and working memory (McCarthy et al., 1994). Overall, the focus of research on the PFC of humans and monkeys in the 1990s was the dorsolateral (granular) PFC and its role in higher cognitive processing.

Perhaps motivated by these findings, publications on the rodent PFC also appeared at an increasing rate starting around 1990 (Fig. 1*A*). Early rodent studies used neurochemical lesions, instead of neuronal recordings, and focused on determining whether rodent PFC areas are crucial for a variety of cognitive processes, such as spatial memory (Shaw and Aggleton, 1993), attention (Muir et al., 1996), and reversal learning (Bussey et al., 1997). Given clear differences in PFC anatomy across species, a debate emerged during this period about whether rodent PFC studies were relevant for understanding the primate PFC, which generally meant the dorsolateral cortex, and especially areas around the principal sulcus in macaques.

Early definitions of prefrontal cortex in rodents were based on a classic study by Rose and Woolsey (1948), who argued that prefrontal areas should be defined as having connections with the mediodorsal nucleus of the thalamus (MD). This view was supported by anatomic studies such as Krettek and Price (1977), Groenewegen (1988), and Condé et al. (1990) and served as the basis for early claims that the rat prefrontal cortex had functions similar to the primate dorsolateral PFC and was crucial for spatial working memory (Kolb, 1984, 1990). However, more recent studies challenged both the anatomic and behavioral bases of this view.

For example, the efferent projections of the thalamic nucleus MD include the “medial precentral area” (Groenewegen, 1988), also known as “medial agranular cortex” or “M2.” In contrast to the prelimbic and infralimbic areas, the medial precentral area was found to receive inputs from sensorimotor parts of the cerebral cortex (Reep et al., 1990; Van Eden et al., 1992; Condé et al., 1995), project to sensorimotor subcortical regions (Reep et al., 1987), and produce movements of the eyes, neck, and jaws when electrically stimulated (Neafsey et al., 1986). As such, it became thought of as potentially analogous to the frontal eye fields of macaque monkeys (Reep et al., 1987) and raised problems for the PFC definition of Rose and Woolsey.

Nucleus MD also projects to orbital regions of the frontal cortex, areas denoted as MO, VO, and LO (Groenewegen, 1988). The connectivity of these regions were studied in monkeys and rats by Price and colleagues (An et al., 1998; Bandler et al., 2000; Floyd et al., 2000, 2001). In a review by Ongür and Price (2000) on anatomically defined networks within the frontal cortex, the “rat” orbital areas were held out of a proposed “orbital network” (based on patterns of connectivity in monkeys and humans) because of the connectivity of the rat areas with “visceral” structures such as the hypothalamus and periaqueductal gray. Such connections are not associated with the primate OFC or dorsolateral PFC, raising more problems for the PFC definition of Rose and Woolsey.

In parallel with these anatomic studies, a number of behavioral studies emerged that challenged the idea that the rodent PFC cortex mediated spatial working memory (Kolb, 1984). Lesions of the prelimbic cortex were found to impair spatial delayed alternation in the T-maze (Brito et al., 1982) and operant chambers (van Haaren et al., 1988; Dunnett et al., 1999). However, these effects were transient in nature (Delatour and Gisquet-Verrier, 2001) and may have been due to reductions in spontaneous alternation (Delatour and Gisquet-Verrier, 1996). Moreover, lesions of mPFC did not impair performance in another test of spatial working memory, the radial arm maze (Delatour and Gisquet-Verrier, 1996; Ragozzino et al., 1998; Gisquet-Verrier and Delatour, 2006). Together, these findings led to the idea that the rodent PFC is important for “working-with-memory” but not spatial memory per se (Moscovitch and Winocur, 1992).

To address the emerging issue of how the frontal cortex of rodents related to the prefrontal cortex of primates, Preuss (1995) wrote a thoughtful review on cross-species issues and concluded that rodents “probably do not provide useful models of human dorsolateral frontal lobe function” but “might prove valuable” for understanding other human frontal areas, such as the cingulate and premotor cortices. Several reviews by anatomists and behavioral neuroscientists appeared several years after the publication of Preuss’ manuscript (Brown and Bowman, 2002; Heidbreder and Groenewegen, 2003; Uylings et al., 2003; Dalley et al., 2004) and argued for common functions, as opposed to common structures, among rodent and primate PFC areas. As a consequence of these competing views, the meaning of the word “prefrontal” in rodent studies remained unclear. However, since the publication of Preuss’s article, one clear change is apparent: the disappearance of the rodent orbital areas (MO, VO, and LO) from the prefrontal taxonomy. A major driver for this change might have been a study by Schoenbaum et al. (1998) that reported data from this part of the rat cortex as some of the first evidence for the encoding of expected outcomes. These researchers referred to the cortical region as “OFC” and this term has largely been used since that time.

In recent years, two lines of research have emerged that may eventually solve the dilemma of what is prefrontal cortex in rodents. Both are based on measures of connectivity and not function. One was developed by Rogier Mars and colleagues using various measures of functional and structural connectivity (Mars et al., 2013, 2016; Neubert et al., 2015). The most recent platform for this approach is capable of revealing common patterns of whole brain connectivity in monkeys and humans (Mars et al., 2018) and might be an excellent approach for understanding the problem of the rodent PFC. Additionally, a tract-tracing approach was developed by Heilbronner et al. (2016), focused on corticostriatal connectivity, and revealed clear common patterns of connections for cortical regions such as the prelimbic cortex.

In parallel with these anatomic findings on the PFC, new insights into PFC function emerged as methods for chronic multi-electrode recordings in behaving rodents emerged in the late 1990s. These methods allowed for examining the functional properties of PFC neurons in rodents performing presumed PFC-dependent behavioral tasks. A hunt for “Goldman-Rakic” neurons in rats was undertaken by several labs.

However, no group found evidence for substantial numbers of neurons in the rodent PFC neurons firing persistently over delay periods to encode spatial information (Jung et al., 1998; Baeg et al., 2003). When persistent activity was found, it was associated with behaviors such as holding down a lever (Narayanan and Laubach, 2006) or maintaining stable body position (Cowen and McNaughton, 2007) over a delay period. These signals from the rat PFC were more in line with early studies on the primate ACC (Niki and Watanabe, 1979; who reporting ramping activity over delay periods in an action timing task) than on the dorsolateral PFC. As a result, many rat researchers shifted away from studying spatial processing (with some exceptions; Spellman et al., 2015), to issues associated with the primate ACC and OFC, such as sequencing actions and outcomes (monkey: Procyk et al., 2000; rat: Lapish et al., 2008), performance monitoring and adjustment (monkey: Ito et al., 2003; rat: Narayanan and Laubach, 2008), and encoding changes in reward contingencies during learning (monkey: Tremblay and Schultz, 1999; Wallis and Miller, 2003; Rudebeck et al., 2013; rat: Schoenbaum et al., 1999; Takahashi et al., 2009; van Duuren et al., 2009; Sul et al., 2010).

**Figure 2.**
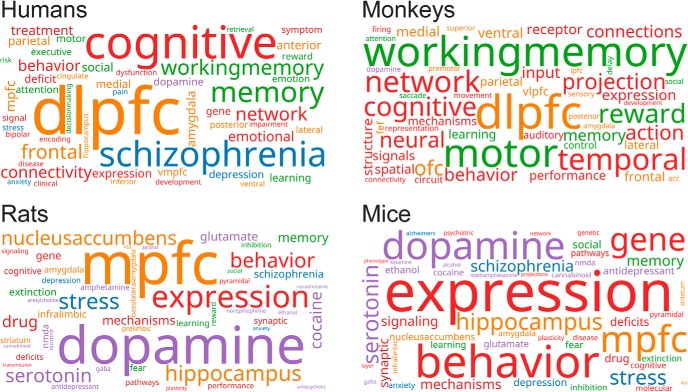
Research focus of prefrontal publications. Word clouds with functional color grouping for papers published on the PFC of mice, rats, monkeys, and humans since 2000. Pharmacological terms are purple. Anatomic terms are orange. Terms associated with diseases and other medical conditions are blue. Psychological constructs are green. Other terms are red.

During the late 2000s, there was a sudden increase in publications on the mouse PFC (Fig. 1*A*). This trend was likely driven by advances in genetic models of human mental illness (Sigurdsson et al., 2010) and new research tools such as optogenetics (Covington et al., 2010). Given recent publication rates (Fig. 1*B*), there are projected to be more studies published on the mouse PFC than the rat PFC by the end of the current decade. We wondered whether some of the same issues that plagued early PFC studies in rats had reemerged in the mouse literature (e.g., claims that mouse PFC is dlPFC and has a role in spatial working memory). We conducted a meta-analysis of recent publications on the PFC in mice, rats, monkeys, and humans by examining word frequencies in articles published since 2000. Word clouds for the most common terms in the abstracts of these articles are shown in Figure 2. Terms are color coded by meaning (pharmacological terms in purple; anatomic terms in orange; diseases and medical conditions in blue; psychological constructs in green; other terms in red). The word clouds show that research on the PFC of primates and rodents is focused on different issues. Human and monkey PFC research emphasizes the dorsolateral PFC, cognitive issues such as working memory, and diseases such as schizophrenia. By contrast, rat and mouse PFC research emphasizes the medial PFC, dopamine, and gene expression. Our meta-analysis suggests that the word “prefrontal” really does mean different things to human and rodent researchers.

**Figure 3.**
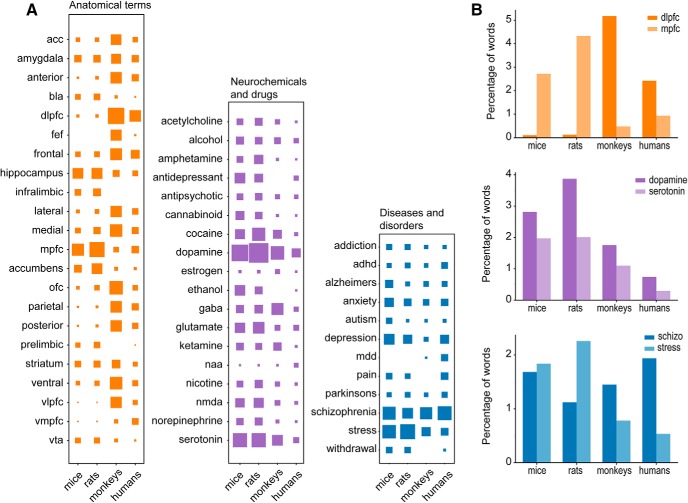
Word frequencies across species. ***A***, Hinton plots for anatomic, neurochemical, and disease-related terms in papers focused on the prefrontal cortex of mice, rats, monkeys, and humans. In each plot, columns denote species and rows denote terms. The relative frequency of each term is represented by a square, and the size of the square is defined as the word count divided by total words. Plots are color coded using the same colors as in Figure 2. Most notable, was the use of certain anatomic terms in monkey studies (e.g., FEF, OFC, vlPFC) that are not common in the human or rodent literature. ***B***, Bar plots of the most common terms for rodents (mice and rats) and primates (monkeys and humans). Anatomic terms were sharply divided across orders (rodents versus primates). Rodent studies were focused on the “mPFC,” and primate studies were focused on the “dlPFC.” By contrast, publications focused on neurotransmitters such as dopamine and serotonin were more common in rodent studies, but the relative frequencies of these terms were not discordant across species. A somewhat different finding was apparent in relative word frequencies for diseases and disorders. Rodent studies, especially in rats, more often addressed stress than studies in primates, and primate studies more often addressed schizophrenia compared to the rodent literature.

Differences in word frequencies within a species are easy to see in word clouds. However, word clouds do not allow so readily for comparing word frequencies across species, as the viewer has to search for a given term in each word cloud and then compare the size of each word across a series of figure panels. A more direct method for comparing terms across species is to plot the relative frequency of each term using Hinton plots, which were developed for visualizing weights of hidden units in neural networks (Hinton and Shallice, 1991). To make Hinton plots of the word frequencies for each functional category of terms, we determined what words were the ten most common in the collection of mouse papers and then added in new terms that were included in the ten most common terms from the other species. This approach revealed that anatomic terms were the most diverse, with 22 terms needed to account for all four species. By contrast, disease-related terms were least diverse, with just 12 terms needed to account for the ten most common terms across species. These reduced list of leading terms (12–22) were then used for the Hinton plots, and those for anatomic, neurochemical, and disease related terms are shown in Figure 3*A*.

The plots revealed interesting insights into the use of specific terms within and among species. For example, several anatomic terms were quite common in the monkey literature (e.g., FEF, OFC, parietal, vlPFC) and not so common in the other species, including the human PFC literature. In a similar way, the common rodent terms prelimbic and infralimbic were largely absent from the monkey and human literature. The most common term in rodents was “mPFC” and the most common term in primates was “dlPFC.” The discordance in these terms was further emphasized in a new bar plot shown in Figure 3*B*, top plot.

The situation with neurochemicals (transmitters and drugs) was less discordant. Some exceptions were dopamine (a bit overemphasized in rodent studies), ethanol but not alcohol (ethanol overemphasized in rodent studies, alcohol equally common across species), and N-acetylaspartic acid (NAA) only occurring in human studies, where it has been used as a marker for neurodegenerative disorders. The two most common neurochemical terms, dopamine and serotonin, showed similar overall relative frequencies across species, with the exception of dopamine. It has been used more in studies of the rat PFC than other species, including in comparison to the mouse literature. This point is depicted in a new bar plot in Figure 3*B*, middle plot.

Usage of terms associated with diseases and disorders was the least diverse across species. Yet, two terms showed up with divergent frequencies in rodents and primates. Schizophrenia is a major term in all species and, perhaps not surprisingly, especially in studies of the human PFC. By contrast, stress was the most common disease-related term in the rat literature, perhaps due to the utility of classic behavioral assays such as restraint and the forced swim test in rodents. The relative frequencies of these terms were further emphasized in a new bar plot in Figure 3*B*, lower plot.

## PFC Nomenclature in Rodents

Rodent PFC is agranular in most species and there is evidence for homologies, based on cytoarchitecture, among medial parts of the PFC, especially the anterior cingulate cortex (ACC), in rodents, humans, and monkeys (Vogt and Gabriel, 1993; Vogt et al., 2013; Vogt and Paxinos, 2014). There have been numerous publications on the medial PFC in humans (3652 publications according to a PubMed search in June 2018, with some researchers referring to the area as ventromedial prefrontal or ventromedial frontal cortex) and rodents (4811 publications). Yet, confusion and controversy about the relevance of these rodent studies has persisted. We think that controversy about rodent PFC has persisted for three reasons, all of which reflect confusion about anatomic nomenclature and not actual physical or physiologic issues. First, the core areas that compose the rodent PFC are characterized using two different nomenclatures (mPFC and ACC). Second, multiple terms and acronyms are used in prominent brain atlases, and the boundaries for the named areas are inconsistent across atlases. Third, hybrid terms, such as prelimbic medial prefrontal cortex, have recently emerged in the literature, which are not used in human or non-human primate studies. We discuss these issues below and argue that these issues have led to limited collaboration between rodent and human researchers, evidenced by the small number of publications done in the same behavioral task across species.

### Dual citizenship

Rodent studies are considered to be “prefrontal” when they report data from three cytoarchitectonically defined parts of the frontal cortex: the prelimbic, infralimbic, and anterior cingulate areas. Studies in humans and monkeys consider these regions to be part of the mPFC, which also includes Walker’s area 10 (Walker, 1940) and the medial parts of area 9, which do not exist in rodents (Semendeferi et al., 2001). A second scheme for cortical nomenclature considers the three rodent medial frontal areas as part of the anterior cingulate cortex (Vogt and Gabriel, 1993). As such, two distinct anatomic terms, ACC and mPFC, can be used to describe the same three cortical regions. Previous versions of stereotaxic atlases (Paxinos and Watson, 1982; Zilles, 1985) referred to the more dorsal and caudal cingulate areas as being the cingulate cortex, while using the terms “prelimbic” and “infralimbic” to describe the rostral medial wall regions of the cortex, which may explain why some rodent studies report functional differences between the ACC and mPFC (Seamans et al., 1995). As our survey described below revealed, it seems that some rodent PFC researchers are aware of, or seemingly prefer, one naming scheme and not the other. This issue has been a major factor in the lack of clarity about the rodent PFC, and it is confusing to non-PFC researchers.

### Alphabet soup/lines in the sand

**Figure 4.**
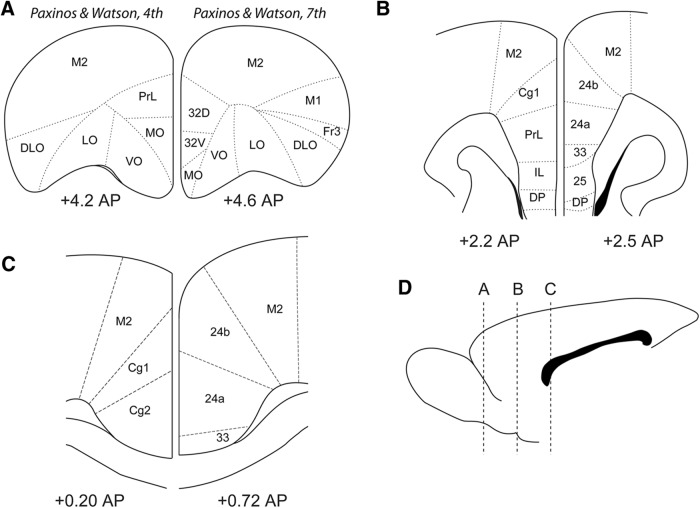
Anatomic terms for rodent PFC areas. ***A***, Sections from the 4th (1998) and 7th (2014) editions of the commonly used Paxinos and Watson rat brain atlas are shown for the most rostral part of the rodent PFC. The 4th edition of the atlas characterized the medial PFC areas as the prelimbic (PrL) and medial orbital (MO) areas. These terms are still used in many recent publications. The 7th edition revised these regions using terms based on Brodmann’s numbers, as dorsal and ventral parts of area 32, but the medial orbital term was retained. ***B***, Sections from the middle parts of the rodent PFC contain more distinct cytoarchitectural areas. Anterior cingulate (Cg1), PrL, and infralimbic (IL) were used in the 4th edition of the atlas. Areas 24 (a and b), 33, and 25 were used in the 7th edition. ***C***, Sections from the most caudal level of the rodent PFC, just anterior to bregma. The areas denoted as Cg1 and Cg2 were relabeled as areas 24a and 24b, respectively, and a new region (to rodent atlases) “area 33” emerged. ***D***, The locations of the sections in ***A–C*** are depicted in a midsagittal section. Please note that it was not possible to use atlas sections at the same anterior-posterior locations due to changes in the content of the editions of the atlases. For example, the 4th edition did not include a section at +4.6 AP and the 7th edition did not include a section at +4.2 AP.

Brain atlases have not made it any easier to define the rodent PFC. Anatomists have used cytoarchitectural criteria to describe medial parts of the rodent frontal cortex as “cingulate cortex” in leading brain atlases (Zilles, 1985; Paxinos and Watson, 1986, 1998; Swanson, 1992). However, a wide range of terms and acronyms have been used to describe these areas. For example, three different acronyms have been used to describe the prelimbic cortex: PL, PrL, and Cg3 (Fig. 4). Similar to our comments above about the use of the word “prefrontal” across species and dual membership in the mPFC and ACC nomenclatures, it would be ideal if a single set of anatomic terms, and associated acronyms, were used to define these cortical regions. The most recent version of the commonly used Paxinos and Watson (2014) rat atlas stated “The nomenclature of the rodent cingulate cortex and related areas has been a major problem for some decades” (page XV). Both mouse (Paxinos and Franklin, 2012) and rat (Paxinos and Watson, 2014) atlases were revised in collaboration with a leading cingulate cortex anatomist, Brent Vogt (Vogt and Gabriel, 1993; Vogt et al., 2013; Vogt and Paxinos, 2014), to use slightly different anatomic boundaries along the medial wall cortex and label areas with Brodmann’s numbers, e.g., area 32 instead of PL and area 24 instead of Cg1 (Fig. 4). The idea behind these changes, according to the authors, was to make the anatomy more in line with studies in primates (humans and monkeys). Unfortunately, a PubMed search reveals that the latest editions of the Paxinos atlases (Paxinos and Franklin, 2012; Paxinos and Watson, 2014) have not been used in many recent studies, and labs still use terms from earlier versions of the Paxinos atlases (e.g., 279 publications with “prelimbic” and four papers with “area 32” in the title of studies using rats or mice, based on a PubMed search in October, 2018).

It is also worth noting that there are two different sets of rodent atlases which stem from either the Paxinos or Swanson initial rat atlas. The terminology and structures found in Paxinos and Franklin (2012) mouse atlas are based off of Paxinos and Watson (1998) rat atlas, while the Allen Brain mouse reference atlas is largely based off of the Swanson (2004) rat atlas (see Appendix 3 in Allen Institute for Brain Science, 2008; Lein et al., 2007). Therefore, differences in rodent PFC nomenclature are often due to using either the Paxinos or Swanson atlases.

### Obfuscation, beclouding, and abstrusity

The three terms in the subheading of this section are synonyms for a lack of clarity in writing. At least they are single words. In recent years, two sets of hybrid terms have emerged in the recent literature (since 2005) for denoting prefrontal regions in rodents. These terms combine the names of the cortical fields, prelimbic and infralimbic, with the term prefrontal cortex, e.g., “prelimbic prefrontal cortex” or “prelimbic medial prefrontal cortex.” Together with the various terms and acronyms described above for the Paxinos and Swanson atlases, it is clear that there are too many terms being used to describe the prefrontal parts of the rodent cortex. We hope that these alternative terms can be eliminated from future publications, as they are redundant, do not add clarity, and make it more difficult to track new findings and incorporate them into the existing literature.

### Why can’t we be friends?

The anatomic issues described above have made it difficult for researchers with expertise on different species to compare their data and, with only a few exceptions (Narayanan et al., 2013; Parker et al., 2015; Hyman et al., 2017), rodent research groups have not collaborated with human or monkey labs to understand medial PFC function. When such collaborations have been done, it has been difficult to agree on how to characterize the rodent areas in relation to medial areas of the human mPFC. For example, in a study done through collaboration between rodent and human researchers (Narayanan et al., 2013), data from the prelimbic area were characterized as “medial frontal cortex” instead of the ACC or mPFC. Medial frontal cortex has not been commonly used to describe this area in the literature, with just 48 papers (<1% of all medial PFC papers) published using the term for rodent studies.

In summary, the question “What, if anything, is rodent prefrontal cortex?” is not easy to address due to major confusion about the anatomic terms that are commonly used to define the prefrontal cortex in rodents. These issues should be resolved as open-source brain atlases, such as the Allen Brain Atlas, are being developed and are becoming the dominant mode of reporting anatomic results for studies on the rodent (mouse) brain.

## Crowdsourcing the PFC

We recently conducted a crowdsourcing effort to assess usage of anatomic terms for the rodent prefrontal cortex. We reached out directly via email to several leading human, monkey, and rodent PFC researchers and also requested via social media for anyone with an informed opinion on rodent PFC to fill out a survey on the topic. In brief, the survey questions inquired about the functional and anatomic extent of the rodent PFC and the specifics of anatomic terms within PFC. We also allowed survey responders to input their confidence levels on each given topic or question. We received 38 survey responses from a wide range of PFC researchers. Even with this small, self-selected sample of opinions, the survey revealed that there is little consensus among researchers about what constitutes the rodent PFC and what terms should be used to describe this cortical region. Three major issues that emerged from the survey are described below.

### What is the prelimbic cortex?

**Figure 5.**
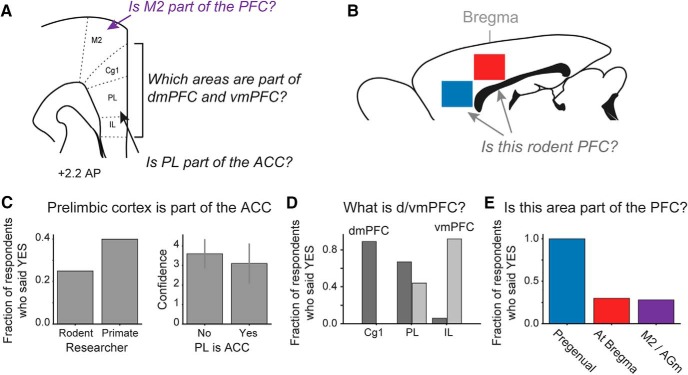
Crowdsourcing the rodent PFC. ***A***, ***B***, 38 respondents were asked questions about how different cytoarchitectural areas fit into the concept of rodent PFC. Major questions included “Is the prelimbic cortex part of the ACC?,” “Which areas comprise the dorsomedial and ventromedial PFC?,” “Is M2 part of the PFC?,” and if two distinct levels of the medial wall cortex are part of the PFC (located anterior to the genu of the corpus callosum, blue box in ***B***; located below bregma, red box in ***B***). ***C***, Only 8 of 32 (25%) respondents working with rodents said that the prelimbic cortex is part of the ACC. Eight of 20 (40%) respondents working with primates answered this question positively. No differences were apparent in the respondents’ confidence in this answer. Given the small sample size, the difference between rodent and primate researchers was not significant (proportions test: χ^2^ = 0.69, df = 1, *p* = 0.4058). This outcome was surprising given that the prelimbic cortex has been considered as a core ACC region (Vogt and Gabriel, 1993). ***D***, There was almost universal agreement that Cg1 is part of the dmPFC (32 of 36 said yes) and IL is part of the vmPFC (33 of 36 said yes). Respondents were divided about how to characterize PL, with 24 of 36 saying it is part of dmPFC and 16 of 36 saying it is part of vmPFC (six respondents included PL in both dmPFC and vmPFC!). ***E***, All respondents agreed that the pregenual region (blue box in ***B***) is part of the rodent PFC. Roughly 25% of respondents felt that the medial wall cortex at bregma (red box in ***B***) and the secondary motor cortex (M2) are PFC regions.

We asked respondents whether the prelimbic area is part of the anterior cingulate cortex in rodents (Fig. 5*A*). Surprisingly, a minority of respondents indicated that the prelimbic cortex is part of the ACC (Fig. 5*C*), despite publications by anatomists that support that designation (Vogt and Gabriel, 1993; Vogt et al., 2013; Vogt and Paxinos, 2014). The prelimbic area has been perhaps the most studied part of the rodent PFC, and we were shocked by this outcome of the survey. One respondent wrote: “Depends on whose definition of the ACC is used.” We are not aware of anatomic studies or reviews on the ACC that do not include the prelimbic cortex (also known as area 32) as a core component of the ACC (e.g., the first anatomic collection dedicated to the ACC: Vogt and Gabriel, 1993). The relevance of the prelimbic area as part of the ACC, i.e., pregenual ACC (pACC), is discussed below.

### dmPFC versus vmPFC

These acronyms are used to describe data from the dorsal and ventral parts of the mPFC. These terms were first used, to our knowledge, in some of the first reversible inactivation and multielectrode recording studies on this part of the rat frontal cortex (Narayanan and Laubach, 2006). More recently, they have been commonly used in studies of the mouse PFC. To assess how researchers use the terms dmPFC and vmPFC, we included questions in our survey about the terms (Fig. 5*A*). Respondents were evenly split on what areas comprise the dmPFC and vmPFC (Fig. 5*D*), with one in six respondents actually including prelimbic cortex in both areas. (A basis for this might be anatomic studies, Gabbott et al., 2005, which have reported differences in anatomic connections of the dorsal and ventral aspects of the prelimbic cortex.) Rather than using these confusing terms (dmPFC and vmPFC), we suggest that research articles state exactly where the experiment was done (e.g., dorsal half of prelimbic cortex instead of dmPFC).

### Rostral-caudal limits on the mPFC

There may be a differing perception of the anatomic extent of rodent mPFC on a rostral to caudal basis. Figure 5*B* shows two areas that have been characterized as medial PFC in the literature. One area is located anterior to the genu of the corpus callosum (prelimbic cortex, blue). The other area is within the cingulate cortex (red), directly below a major skull landmark used for stereotaxic surgeries in rodents (bregma). All respondents said that the rostral region is mPFC (Fig. 5*E*). Additionally, 25% said that the caudal region is mPFC. This is surprising given that the caudal area is immediately adjacent to the primary motor cortex for the forelimb (Donoghue and Wise, 1982) and is considered as part of the mid, not anterior, cingulate cortex (Vogt, 2016).

### Lateral limits of the rodent PFC

The area immediately dorsal and lateral to the classic mPFC/ACC area Cg1 has been called several names (medial precentral cortex, PrCm; medial agranular cortex, AGm, and secondary motor cortex, M2 or MOs). In recent years, data from this area have been characterized as “prefrontal” (Savage et al., 2017). Some researchers have been careful to omit the “pre” from prefrontal when describing this area (Siniscalchi et al., 2016; for review, see Barthas and Kwan, 2017). This is reasonable as several studies have reported projections to muscles controlling jaw and tongue movements (Yoshida et al., 2009) and electrical stimulation producing movements of the whiskers (Brecht et al., 2004), and head/neck (Erlich et al., 2011). The region contains numerous neurons that project to the spinal cord (Li et al., 1990), far more than exist in the adjacent classic PFC regions. When data are obtained from this region, some researchers have referred to the area as medial frontal cortex, as opposed to medial medial prefrontal cortex (Amarante et al., 2017). To assess appreciation of whether this area is part of rodent PFC, we included a question about it in our survey and found that ∼25% of respondents said that M2 is part of the rodent PFC (Fig. 5*E*).

In summary, our survey of prefrontal nomenclature supports our claim that there is confusion and controversy, even among PFC researchers, about what defines prefrontal cortex in rodents. This may be driven by inconsistencies between different rodent brain atlases, between atlases and peer-reviewed anatomic studies and between different PFC-focused labs (e.g., some use mPFC for rostral medial cortex and ACC for caudal medial cortex, others call the whole area mPFC). These issues really need to be addressed to advance our understanding of PFC structure and function in rodents and to motivate collaboration among labs, especially those working in different species.

## Evolutionary Homology: From Flat to Curved Brains

**Figure 6.**
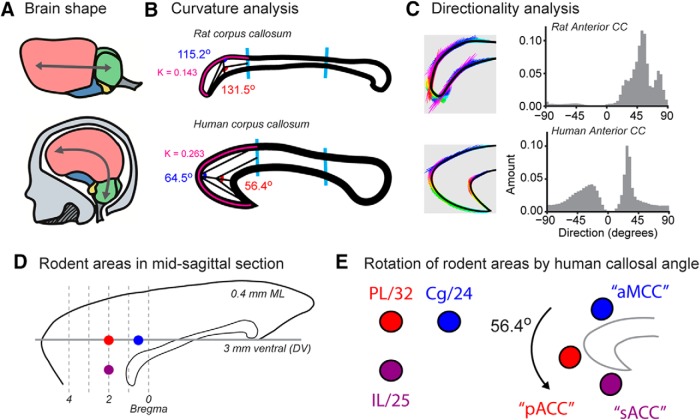
Proposed cross-species homologies. ***A***, Rodent brains are flat. Modern primate brains are curved. ***B***, Quantification of the curvature of anterior corpus callosum (using the Kappa library for FIJI). The point of maximum curvature along the superior edge of the anterior third of the corpus callosum is 1.84 times more curved in humans (K = 0.263) than in rats (K = 0.143). Interior angles with vertex at the point of maximum curvature. The rat callosum has obtuse interior angles. The human callosum has acute interior angles. ***C***, Directionality analysis (using FIJI) of the anterior third of the callosum reveals unimodality in rat with a peak of approximately -52°. The human CC peaks at -26° and 16°, indicative of its parabolic shape. A third peak at ∼54° is due to the internal angle of the rostrum. Images used for the directionality analysis are shown on the left and directions are encoded with false color (rainbow colormap with red equal to -90° and violet equal to +90°). ***D***, Locations of three main parts of the rodent PFC (Cg1, blue; PL, red; IL, purple) shown on a midsagittal section (0.4 mm ML). ***E***, Rotation of the three points representing each cortical region by the measured curvature of the human callosum shifts the rodent PFC areas into approximate locations associated with the major divisions of the human medial frontal cortex. This analysis suggests that (1) the area called Cg1 in rodents may be homologous to the anterior part of the midcingulate cortex (aMCC) in primates, (2) the area called PL in rodents may be homologous to the called pACC (pregenual ACC) in primates, and (3) the area called IL in rodents may be homologous to the area called sACC (subgenual ACC) in primates.

We would like to propose an alternative way to think about rodent PFC, which may help with incorporating findings across species into an integrated field of PFC research. Our proposal is based on a major difference between the brains of rodents and primates, putting size aside for the time being. In rodent brains, points from rostral to caudal are contained within a horizontal plane. In contrast, extant primate brains are curved, i.e., concave from front to back (Fig. 6*A*). However, extinct basal primates, known as plesiadapiforms, had linear brain plans similar to living rodents (Long et al., 2015). The consequences of these gross changes in brain shape may provide a clue for relating medial PFC regions across species. A similar mechanism was proposed by Vogt and Paxinos (2014), where they suggest that the expansion of the human midcingulate cortex (MCC), and the addition of the posterior cingulate cortex (PCC), leads to a displacement of the ACC both rostrally and ventrally around the genu of the corpus callosum. Further evidence for this idea comes from a recent computational and physical modeling study of cortical expansion (Tallinen et al., 2016) that suggested that cortical convolutions arise from compressive stress. In their studies on the formation of gyri, this study found that compressive stress was especially pronounced within the frontal and temporal lobes and that compressive effects are maximum at angles that are perpendicular to the angle of maximum curvature,

In addition to the increase in prefrontal tissue volume and development of granular cytoarchitecture, another species difference is the expansion and curvature of the corpus callosum in primates (Fig. 6*B*,*C*). By measuring the curvature of the primate callosum and applying it to points in space representing the three main parts of the rodent medial PFC, we found that the three rodent areas rotate to the locations associated with the three major functional zones of the primate ACC: the subgenual region (sACC), the pregenual region (pACC), and the mid-anterior cingulate cortex (MCC; compare our Fig. 6*E* with Shackman et al., 2011, their Fig. 1*C*; see Table 1). Thinking of rodent PFC in terms of these primate regions of the ACC should help clarify cross-species findings and might motivate human and monkey researchers to add simple behavioral tasks to their studies so that results can be better related across species.

**TABLE 1.** Terms and acronyms used to describe prefrontal areas in humans, monkeys, and rodents

Human/monkey term	Rodent term (Paxinos atlases, pre-2013)	Rodent term (Swanson, 2004; Allen Brain Atlas)	Brodmann’s term
Midcingulate cortex (MCC)	ACC, Cg1, Cg2	ACAd	Area 24
Pregenual ACC (pACC)	PrL, PL, Cg3	PL	Area 32
Subgenual ACC (sACC)	IL	ILA	Area 25

## Suggestions for Future Studies on the Rodent PFC

We would like to close this perspective with some suggestions for improving the clarity, rigor, and reproducibility of rodent PFC research. First, we recognize the difficulty in convincing an entire field to change their preferred terminology for rodent PFC. To reduce confusion rodent researchers should at least clarify the precise anatomic location (e.g., when reporting data from neural recordings, use precise terms such as “prelimbic” instead of the more generic “ACC” or “mPFC” and/or estimated distances from landmarks such as bregma, e.g., 3.2 mm) of their data in publications on rodent prefrontal cortex, should always report specific coordinates used in surgery (along with along with information on the strain, sex, and age of subjects), and should always provide images showing locations of experimental probes and viral fields for single animals and in group summaries. Second, hybrid terms such as “infralimbic medial prefrontal” should not be used, and the community (especially journal editors and manuscript reviewers) should speak up when such unnecessary and confusion-generating terms appear in a preprint or manuscript that is under consideration for publication. Third, and most important, there is a serious need for community engagement to address the lack of clarity and resulting confusion generated by rodent PFC researchers not having adopted strict standards for anatomic details. This might best be addressed through a dedicated workshop to foster consensus on the use of precise anatomic terms for the rodent PFC, involving leading rodent PFC research labs, expert neuroanatomists, funding partners (NIH), foundations with a vested interest in rodent neuroanatomy (e.g., Allen Brain), and editors from leading neuroscience journals.

## Information on Meta-Analysis, Survey, and Anatomic Measurements

### PubMed citations, word frequencies, and word clouds

PubMed was searched for publications on the prefrontal cortex in humans, monkeys, rats, and mice using search terms such as “(prefrontal cortex) AND (primate[tiab] OR monkey[tiab])” between the years 2000 and 2017. Timelines for the plots in Figure 1*A* were saved into CSV files and plotted using Python code. Publication counts were removed for 2017, given apparent incomplete posting of all papers to PubMed for this most recent complete year. Word clouds were generated for these searches using the freely available reference manager Zotero (https://www.zotero.org/).

Bibliographies were created for each set of search terms using lists of PMIDs, imported into Zotero using the “Add items by identifier” tool, and saved in CSV (comma-separated values) formats. Custom-written Python code was used to select up to 10,000 of the most recent publications for each species. Then, abstracts were extracted and the resulting text was filtered to remove common stop words (a, the, and, etc.); common science terms (experiment, hypothesis, subjects, etc.); and species-specific words (monkey, rat, mouse, etc.). The remaining text was then processed in R using the text-mining library (‘tm’) to create matrices of words and number of appearances. Results were then manually sorted into functional categories in Excel: neurochemical terms (neurotransmitters and drugs), anatomic terms (regions of pfc and other brain areas), disease-related terms (diseases, disorders, and conditions), psychological functions (associated function), and other terms (e.g., gene and expression). The matrix of sorted words and their frequencies were then imported into Python, where word clouds were made using the word cloud library (https://github.com/amueller/word_cloud).

Dictionaries were created for each of the functional categories and used to assign common colors to words in each category.

### Survey on the rodent PFC

The survey consisted of questions about the inclusion of various parts of the rodent frontal cortex as “prefrontal” areas. Images from atlases were used to query the rostral-to-caudal and medial-to-lateral limits of the prefrontal cortex and to clarify inclusion into the dorsomedial and ventromedial PFC in rats and macaque monkeys. The survey was sent to attendees of the Computational Properties of the Prefrontal Cortex (CPPC) conference and was also advertised via Twitter. For data analysis, responses were only considered if the respondent has been first or last author on at least one journal article on the prefrontal cortex. Survey results were collated into a spreadsheet and analyzed using a custom-written Jupyter notebook using the following Python libraries: numpy, matplotlib, seaborn, and pandas. Statistical analysis of results was conducted in R, and were done in the same Python notebook using the rpy2 library. Survey results and analysis/plotting code are available on request of the corresponding author.

### Participants

Linda Amarante, Bruno Averbeck, Mark Baxter, Sebastien Bouret, Kevin Braunscheidel, Hannah Clarke, Michael Cole, Ilka Diester, Fabricio Do Monte, David Euston, Joshua Gordon, Sarah Heilbronner, Cyril Herry, Clay Holroyd, Nicole Horst, James Hyman, Sara Keefer, Christoph Kellendonk, Alex Kwan, Ryan LaLumiere, Christopher Lapish, Martin Pare, David Moorman, Nandakumar Narayanan, Nate Powell, Emmanuel Procyk, David Redish, Trevor Robbins, Peter Rudebeck, Jerome Sallet, Jeremy Seamans, Melissa Sharpe, Michael Siniscalchi, Elena Vazey, Mark Walton, Charlie Wilson, Steve Wise, and John Woodward

### Analysis of callosum structure in humans and rats

The human callosum was obtained from Aboitiz and Montiel (2003). The rat callosum was obtained from Paxinos Atlas 6th edition. Directionality of the outer edge of the anterior third was assessed using the Directionality package in FIJI (https://imagej.net/Directionality).

Curvature of the dorsal edge was analyzed using the Kappa package in FIJI (https://github.com/brouhardlab/Kappa/). Angles were calculated using the point of highest curvature as the vertex. Both the angle using points along the outer edge of the callosum and the angle using the midpoint of the width of the callosum were calculated in FIJI.

## Synthesis

Reviewing Editor: Mark Baxter, Mount Sinai School of Medicine Decisions are customarily a result of the Reviewing Editor and the peer reviewers coming together and discussing their recommendations until a consensus is reached. When revisions are invited, a fact-based synthesis statement explaining their decision and outlining what is needed to prepare a revision will be listed below. The following reviewer(s) agreed to reveal their identity: Alicia Izquierdo.

Thank you for submitting your paper “What, if anything, is rodent prefrontal cortex?” to eNeuro. I have consulted two reviewers for their comments on the manuscript. We are all enthusiastic about the meta-analytic approach and the illustration of confusion surrounding rodent prefrontal cortex. We have a few changes to suggest to the paper, but they should be straightforward to address. I am separating them into more substantive comments and minor word choice/editing.

Substantive:

- As far as PFC nomenclature goes, Figure 1 Uylings et al. (2003) showed the extent for what different groups considered PFC in rat until that time. Krettek and Price (1977) included LO and AIv and later Groenenwegen (1988) expanded to include VO MO. It wasn't until Preuss (1995) that these areas disappeared from PFC taxonomy. Yet I could find no mention of this in the manuscript, and I think LO AIv and perhaps also VO and MO deserves some attention.

- I do think Vogt and Paxinos (2014) could be credited a bit more for the curvature idea presented in this manuscript, since they have previously offered a mechanism (expansion of midcingulate cortex) in their 2014 paper comparing mouse, rat, and human: “The substantial expansion of MCC and addition of PCC in the human results in a major displacement of ACC rostrally and ventrally around the genu of the corpus callosum.”

- It would be useful to have more description of the methodology used to generate the word clouds. How did the meta-analysis capture all of the rodent articles if so many anatomical terms are in common use - were multiple search terms used? And if so, what were they? Did the word clouds include entire articles, abstracts or just titles? How were terms grouped into clusters of ‘anatomical’, ‘disease and medical’, etc.?

Minor:

- If possible, it would be nice to see a monkey word cloud.

- In the word clouds, the different themes across species must be largely due to different tools available in each. I think the authors' point is how this leads to differences in ideas and theories, but their views here aren't fully spelled out. It might even be interesting to exclude technique-driven words (e.g. gene / connectivity) or directly compare frequencies of the same words across species, to get a sense of the conceptual differences in the fields.

- The NCBI web link appears to be broken.

- The authors write in the introduction, “For instance, it is not uncommon for researchers to use information about one prefrontal area in macaque monkeys (or humans) in an attempt to understand a completely different prefrontal area in a rodent species.” A citation is not necessary, but it would be great to have an example of such an instance.

- There should be some mention somewhere that the labels we use are based on cytoarchitectonic subdivisions.

- The authors do a great job of pointing out different criteria for what could be considered homology, “common functions, as opposed to common structures,” but there is another approach based on connectivity with subcortical structures- Heilbronner et al. Biol Psychiatry 2016; that the authors may choose to cite.

- “.. our meta-analysis reveals that rodent researchers have not focused on cognitive functions associated with lateral, but not medial, PFC.” This is a double-negative, awkward phrasing.

- “As a result, many rat researchers shifted away from studying spatial processing to issues associated with the ACC, such as sequencing actions and outcomes (monkey: Procyk et al., 2000; rat: Lapish et al., 2008) and performance monitoring and adjustment (monkey: Ito et al., 2003; rat: Narayanan and Laubach, 2008).” Are there other such examples that the authors can pull from in other PFC subregions across monkey and rat? As currently presented, the manuscript is quite ACC-centric.

- “there should be more studies published on the mouse PFC than the rat PFC by the end of the current decade.” The way this is written makes it sound more advisory than a prediction, suggest “there are projected to be more studies...” or some such phrasing.

- Top of page 10, there is the assertion that authors typically don't provide enough information on location of PFC targets. But how often are stereotaxic coordinates not included, or representative images not included? I would say this is fairly rare and likely not to contribute to the confusion/controversies.

## Author Response

Thank you for submitting your paper “What, if anything, is rodent prefrontal cortex?” to eNeuro. I have consulted two reviewers for their comments on the manuscript. We are all enthusiastic about the meta-analytic approach and the illustration of confusion surrounding rodent prefrontal cortex. We have a few changes to suggest to the paper, but they should be straightforward to address. I am separating them into more substantive comments and minor word choice/editing. Thank you very much for this very useful feedback on our manuscript. The clarity of the proposed changes made it easy for us to do revisions, and we believe that the manuscript is much better thanks to the review process. We addressed each of the substantive and minor points that were raised. We also made some minor changes to the manuscript that are described at the end of our response to the reviews. We hope that you will now find the manuscript acceptable for publication in eNeuro.

-The authors Substantive: - As far as PFC nomenclature goes, Figure 1 Uylings et al. (2003) showed the extent for what different groups considered PFC in rat until that time. Krettek and Price (1977) included LO and AIv and later Groenenwegen (1988) expanded to include VO MO. It wasn't until Preuss (1995) that these areas disappeared from PFC taxonomy. Yet I could find no mention of this in the manuscript, and I think LO AIv and perhaps also VO and MO deserves some attention. Thank you for raising this issue. We modified the text in the ‘Publication trends’ section of the manuscript to address this issue. Fairly extensive text was added after the sentence starting “Given clear differences in PFC anatomy across species...” and before the sentence starting “Preuss (1995) wrote a thoughtful review...” to address this issue. The text is shown below: “Early definitions of prefrontal cortex in rodents were based on a classic study by Rose and Woolsey (1948), who argued that prefrontal areas should be defined as having connections with the mediodorsal nucleus of the thalamus (MD). This view was supported by anatomical studies such as Krettek and Price (1977), Groenewegen (1988), and Conde et al. (1990), and served as the basis for early claims that the rat prefrontal cortex had functions similar to the primate dorsolateral PFC and was crucial for spatial working memory (Kolb, 1984, 1990). However, more recent studies challenged both the anatomical and behavioral bases of this view. For example, the efferent projections of the thalamic nucleus MD include the ‘medial precentral area’ (Groenewegen, 1988), aka ‘medial agranular cortex’ or ‘M2’. In contrast to the prelimbic and infralimbic areas, the ‘medial precentral area’ was found to receive inputs from sensorimotor parts of the cerebral cortex (Reep et al., 1990; Van Eden et al., 1992; Conde et al., 1995), project to sensorimotor subcortical regions (Reep et al., 1987), and produce movements of the eyes, neck, and jaws when electrically stimulated (Neafsey et al., 1986). As such, it became thought of as potentially analogous to the frontal eye fields of macaque monkeys (Reep et al., 1987) and raised problems for the PFC definition of Rose and Woolsey. Nucleus MD also projects to orbital regions of the frontal cortex, areas denoted as MO, VO, and LO (Groenewegen, 1988). The connectivity of these regions were studied in monkeys and rats by Price and colleagues (e.g. An et al., 1998; Floyd et al., 2000; Bandler et al., 2000). In a review by Ongur and Price (2000) on anatomically defined networks within the frontal cortex, the ‘rat’ orbital areas were held out of a proposed ‘orbital network’ (based on patterns of connectivity in monkeys and humans) because of the connectivity of the rat areas with ‘visceral’ structures such as the hypothalamus and periaqueductal gray. Such connections are not associated with the primate OFC or dorsolateral PFC, raising more problems for the PFC definition of Rose and Woolsey. In parallel with these anatomical studies, a number of behavioral studies emerged that challenged the idea that the rodent PFC cortex mediated spatial working memory (Kolb, 1984). Lesions of the prelimbic cortex were found to impair spatial delayed alternation in the T-maze (Brito et al., 1982) and operant chambers (van Haaren et al., 1988; Dunnett et al., 1999). However, these effects were transient in nature (Delatour and Gisquet- Verrier, 2001) and may have been due to reductions in spontaneous alternation (Delatour and Gisquet-Verrier, 1996). Moreover, lesions of mPFC did not impair performance in another test of spatial working memory, the radial arm maze (Delatour and Gisquet-Verrier, 1996; Ragozzino et al., 1998; Gisquet-Verrier and Delatour, 2006). Together, these findings led to the idea that the rodent PFC is important for ‘workingwith- memory’ but not spatial memory per se (Moscovitch and Winocur, 1992).

To address the emerging issue of how the frontal cortex of rodents related to the prefrontal cortex of primates, Preuss (1995) wrote a thoughtful review...” In addition to this new text, we also modified this section of the manuscript by concluding with your observation that terms such as MO, VO, and LO “disappeared from PFC taxonomy” and became referred to as ‘OFC’ following an influential paper by Schoenbaum, Chiba, and Gallagher (1998) that provided the first evidence for ‘outcome encoding’ by the rodent OFC.

“As a consequence of these competing views, the meaning of the word ‘prefrontal’ in rodent studies remained unclear. However, since the publication of Preuss's article, one clear change in the literature was the disappearance of the rodent orbital areas (MO, VO, and LO) from the prefrontal taxonomy. A major driver for this change might have been a study by Schoenbaum and colleagues (1998) that reported data from this part of the rat cortex as some of the first evidence for the encoding of expected outcomes. These researchers referred to the cortical region as ‘OFC’ and this term has largely been used since that time.”

- I do think Vogt and Paxinos (2014) could be credited a bit more for the curvature idea presented in this manuscript, since they have previously offered a mechanism (expansion of midcingulate cortex) in their 2014 paper comparing mouse, rat, and human: “The substantial expansion of MCC and addition of PCC in the human results in a major displacement of ACC rostrally and ventrally around the genu of the corpus callosum.” Thank you for bringing this up, and we agree that their figure 6 diagram is a great example of the influence of the corpus callosum in shaping the cingulate cortex. We have added into the text in the paragraph on Evolutionary Homology (pp. 13) to attribute the MCC expansion mechanism to Vogt and Paxinos: “A similar mechanism was proposed by Vogt and Paxinos (2014), where they suggest that the expansion of the human mid-cingulate cortex (MCC) -- and the addition of the posterior cingulate cortex (PCC) - leads to a displacement of the ACC both rostrally and ventrally around the genu of the corpus callosum.”

- It would be useful to have more description of the methodology used to generate the word clouds. How did the meta-analysis capture all of the rodent articles if so many anatomical terms are in common use - were multiple search terms used? And if so, what were they? Did the word clouds include entire articles, abstracts or just titles? How were terms grouped into clusters of ‘anatomical’, ‘disease and medical’, etc.?

Thanks for asking about these details. The original text was underdeveloped. The section on how we made word clouds was expanded using the text below. We only used the term ‘prefrontal’ in the title-or-abstract [tiab] to find papers and so it is possible that we missed some rodent studies that did not denote the study as on the prefrontal cortex. The search strategy is described below as well as the text that was used (abstracts only) and details on how groupings were made (manually). We hope that the expanded text addresses your concerns. PubMed citations, word frequencies, and word clouds: PubMed was searched for publications on the prefrontal cortex in humans, monkeys, rats, and mice using search terms such as “(prefrontal cortex) AND (primate[tiab] OR monkey[tiab])” between the years 2000 and 2017. Timelines for the plots in Figure 1A were saved into CSV files and plotted using Python code. Publication counts were removed for 2017, given apparent incomplete posting of all papers to PubMed for this most recent complete year. Word clouds were generated for these searches using the freely available reference manager Zotero (https://www.zotero.org/). Bibliographies were created for each set of search terms using lists of PMIDs, imported into Zotero using the “Add items by identifier” tool, and saved in CSV (comma-separated values) formats. Custom-written Python code was used to select up to 10,000 of the most recent publications for each species. Then, abstracts were extracted and the resulting text was filtered to remove common stop words (a, the, and, etc.); common science terms (experiment, hypothesis, subjects, etc.); and species specific words (monkey, rat, mouse, etc.). The remaining text was then processed in R using the text-mining library (‘tm’) to create matrices of words and number of appearances. Results were then manually sorted into functional categories in Excel: neurochemical terms (neurotransmitters and drugs), anatomical terms (regions of pfc and other brain areas), disease-related terms (diseases, disorders, and conditions), psychological functions (associated function), and other terms (e.g. gene and expression). The matrix of sorted words and their frequencies were then imported into Python, where word clouds were made using the wordcloud library (https://github.com/amueller/word_cloud). Dictionaries were created for each of the functional categories and used to assign common colors to words in each category. Minor:

- If possible, it would be nice to see a monkey word cloud. Thank you for this suggestion. We made a comparable word cloud for the monkey literature. It is quite revealing, and strongly supports a dichotomy between rodent and primate PFC research. We added the new word cloud to the manuscript. With four word clouds, the original Figure 1 became quite massive. So, we split the figure into two, with the new Figure 1 showing publications trends and the new Figure 2 showing the word clouds.

- In the word clouds, the different themes across species must be largely due to different tools available in each. I think the authors' point is how this leads to differences in ideas and theories, but their views here aren't fully spelled out. It might even be interesting to exclude techniquedriven words (e.g. gene / connectivity) or directly compare frequencies of the same words across species, to get a sense of the conceptual differences in the fields. This is an excellent point. We considered removing technique-related terms such as gene, expression, and connectivity; however, their presence revealed major differences in research foci across species. For example, not surprisingly, expression was more common in publications on mice.

To directly compare frequencies of the same terms across species, we created a new figure (Figure 3) that depicts relative word frequencies in adjacent columns using Hinton plots and in bar plots for select words that were most common overall. The new text below was added to the manuscript to summarize the new plots and findings. Differences in word frequencies within a species are easy to see in word clouds. However, word clouds do not allow so readily for comparing word frequencies across species, as the viewer has to search for a given term in each word cloud and then compare the size of each word across a series of figure panels. A more direct method for comparing terms across species is to plot the relative frequency of each term using Hinton plots, which were developed for visualizing weights of hidden units in neural networks (Hinton and Shallice, 1991). To make Hinton plots of the word frequencies for each functional category of terms, we determined what words were the ten most common in the collection of mouse papers, and then added in new terms that were included in the ten most common terms from the other species. This approach revealed that anatomical terms were the most diverse, with 22 terms needed to account for all four species. By contrast, disease-related terms were least diverse, with just 12 terms needed to account for the ten most common terms across species. These reduced list of leading terms (12-22) were then used for the Hinton plots, and those for anatomical, neurochemical, and disease related terms are shown in Figure 3A.

The plots revealed interesting insights into the use of specific terms within and among species. For example, several anatomical terms were quite common in the monkey literature (e.g. FEF, OFC, parietal, vlPFC) and not so common in the other species, including the human PFC literature. In a similar way, the common rodent terms prelimbic and infralimbic were largely absent from the monkey and human literature. The most common term in rodents was ‘mPFC’ and the most common term in primates was ‘dlPFC’. The discordance in these terms was further emphasized in a new bar plot

shown in Figure 3B (top plot).

The situation with neurochemicals (transmitters and drugs) was less discordant. Some exceptions were dopamine (a bit overemphasized in rodent studies), ethanol but not alcohol (ethanol overemphasized in rodent studies, alcohol equally common across species), and N-Acetylaspartic acid (NAA) only occurring in human studies, where it has been used as a marker for neurodegenerative disorders. The two most common neurochemical terms, dopamine and serotonin, showed similar overall relative frequencies across species, with the exception of dopamine. It has been used more in studies of the rat PFC than other species, including in comparison to the mouse literature. This point is depicted in a new bar plot in Figure 3B (middle plot). Usage of terms associated with diseases and disorders was the least diverse across species. Yet, two terms showed up with divergent frequencies in rodents and primates. Schizophrenia is a major term in all species and, perhaps not surprisingly, especially in studies of the human PFC. By contrast, stress was the most common disease-related term in the rat literature, perhaps due to the utility of classic behavioral assays such as restraint and the forced swim test in rodents. The relative frequencies of these terms was further emphasized in a new bar plot in Figure 3B (lower plot).

- The NCBI web link appears to be broken. The link was to a website called FLink that was discontinued by the NIH during the time when this manuscript was under review. Instructions for using standard PubMed and Zotero instead have been included in the revised manuscript, as described above in our response to the third substantive issue.

- The authors write in the introduction, “For instance, it is not uncommon for researchers to use information about one prefrontal area in macaque monkeys (or humans) in an attempt to understand a completely different prefrontal area in a rodent species.” A citation is not necessary, but it would be great to have an example of such an instance. We were initially reluctant to include references for this point, but understand the request and the need to document our statements. The following studies have been cited in the revised manuscript. In fact, they translated results in the opposite direction suggested by our text, and so we have revised the text in the new version of the manuscript. The studies cited below went from rodent prelimbic cortex to human dlPFC. The first manuscript is a preview article highlighting findings from the second paper, which is a clinical pilot study on the human dlPFC. It was based on findings from the third paper, which was done on the rodent mPFC (prelimbic cortex).

Ferenczi and Deisseroth, 2016 - PMID: 26780510 Terraneo et al., 2016 - PMID: 26655188 Chen et al., 2013 - PMID: 23552889 - There should be some mention somewhere that the labels we use are based on cytoarchitectonic subdivisions. Thank you for pointing this out. A comment on this issue was added on page 9 in the section

“PFC nomenclature in rodents” and on page 10 in the ‘Alphbet soup’ subsection. - The authors do a great job of pointing out different criteria for what could be considered homology, “common functions, as opposed to common structures,” but there is another approach based on connectivity with subcortical structures- Heilbronner et al. Biol Psychiatry 2016; that the authors may choose to cite.

Thank you for mentioning this study, and we agree that it should be included. We also cited a promising approach for cross-species anatomy developed by Rogier Mars and colleagues: e.g. https://elifesciences.org/articles/35237. Text was added immediately after the heavily revised section about the Preuss study (p. 6), and is shown below:

“In recent years, two lines of research have emerged that may eventually solve the dilemma of what is prefrontal cortex in rodents. Both are based on measures of connectivity, and not function. One was developed by Rogier Mars and colleagues using various measures of functional and structural connectivity (Mars et al., 2013; Neubert et al., 2015; Mars et al., 2016). The most recent platform for this approach is capable of revealing common patterns of whole brain connectivity in monkeys and humans (Mars et al., 2018), and might be an excellent approach for understanding the problem of the rodent PFC. Additionally, a tract-tracing approach was developed by Heilbronner et al. (2016), focused on corticostriatal connectivity, and revealed clear common patterns of connections for cortical regions such as the prelimbic cortex.”

- “.. our meta-analysis reveals that rodent researchers have not focused on cognitive functions associated with lateral, but not medial, PFC.” This is a double-negative, awkward phrasing. Thanks for catching this issue. We revised the text as follows: “...our meta-analysis reveals that rodent researchers have focused on cognitive functions associated with medial, but not lateral, PFC.”

- “As a result, many rat researchers shifted away from studying spatial processing to issues associated with the ACC, such as sequencing actions and outcomes (monkey: Procyk et al., 2000; rat: Lapish et al., 2008) and performance monitoring and adjustment (monkey: Ito et al., 2003; rat: Narayanan and Laubach, 2008).” Are there other such examples that the authors can pull from in other PFC subregions across monkey and rat? As currently presented, the manuscript is quite ACC-centric. Thanks for raising this important point. We focused on ACC-related studies given our view that the literature for OFC has diverged and there is no other PFC in rodents. To address this issue, we added some text about the OFC and also a citation to some more recent work that continued to emphasize spatial coding by rodent mPFC/ACC. The text was revised as follows: “As a result, many rat researchers shifted away from studying spatial processing (with some exceptions, e.g. Spellman et al., 2015), to issues associated with the primate ACC and OFC, such as sequencing actions and outcomes (monkey: Procyk et al., 2000; rat: Lapish et al., 2008), and performance monitoring and adjustment (monkey: Ito et al., 2003; rat: Narayanan and Laubach, 2008), and encoding changes in reward contingencies during learning (monkey: Tremblay and Schultz, 1999; Wallis and Miller, 2003; Rudebeck et al., 2013; rat: Schoenbaum et al., 1999; Takahashi et al., 2009; van Duuren et al., 2009; Sul et al., 2010).” CHANGES HIGHLIGHTED IN RED - “there should be more studies published on the mouse PFC than the rat PFC by the end of the current decade.” The way this is written makes it sound more advisory than a prediction, suggest “there are projected to be more studies...” or some such phrasing.

We have changed the phrasing on this to instead say “Given recent publication rates (Figure 1B), there are projected to be more studies published on the mouse PFC than the rat PFC” - Top of page 10, there is the assertion that authors typically don't provide enough information on location of PFC targets. But how often are stereotaxic coordinates not included, or representative images not included? I would say this is fairly rare and likely not to contribute to the confusion/controversies.

Indeed, this point is correct for stereotaxic coordinates. We went back and checked the last 20 papers published on rodent PFC using PubMed and found that all studies reported stereotaxic coordinates and most showed some example of localization of electrodes, optrodes, or infusion cannula. Therefore, we modified the text to focus on the specific issue of the acronyms dmPFC and vmPFC.

“Rather than using these confusing terms (dmPFC and vmPFC), we suggest that research articles state exactly where the experiment was done (e.g. dorsal half of prelimbic cortex instead of dmPFC).” Additional changes:

1. The anatomical term used in Figure 6, and related text on pages 14-15, was changed to denote the region of the cingulate cortex above the corpus callosum as MCC, instead of dACC. The anatomical basis for this change is described in Vogt, 2016.

2. An early motivation for our manuscript was a study by Tallinen and colleagues (2016), which reported evidence for the rotational (or shearing) forces that might have resulted in changes in the shape of the corpus callosum. We omitted this study from the original version of our manuscript, but added a comment about it in the ‘Evolutionary homology’ section (p 14) of the revised manuscript. “Further evidence for this idea comes from a recent computational and physical modeling study of cortical expansion (Tallinen et al., 2016) that suggested that cortical convolutions arise from compressive stress. In their studies on the formation of gyri, this study found that compressive stress was especially pronounced within the frontal and temporal lobes, and that compressive effects are maximum at angles that are perpendicular to the angle of maximum curvature,”

3. We received feedback from the manuscript while it was under review from a mouse researcher who noted we did not consider leading mouse atlases when discussing changes to rodent atlases. We included these changes throughout the manuscript to add in details about the Paxinos and Franklin mouse atlas and more details on the Allen Brain atlas. Interestingly, these atlases were apparently all derived from rat atlases.

4. We became aware of a project called NeuroNames during the period when our manuscript was under review. This project is a database of definitions used for neuroanatomical structures and acronyms that was created with the intention to “resolve ambiguities of neuroanatomical nomenclature”. We added a comment about this project in the first paragraph of the revised manuscript.
